# Physics‐Embedded Neural Network: A Novel Approach to Design Polymeric Materials

**DOI:** 10.1002/advs.202522475

**Published:** 2026-01-28

**Authors:** Siqi Zhan, Hengheng Zhao, Haotian Wang, Zhanjie Liu, Qian Li, Weifeng Zhang, Qingsong Zhao, Liqun Zhang, Jun Liu

**Affiliations:** ^1^ State Key Laboratory of Organic‐Inorganic Composites Beijing University of Chemical Technology Beijing P. R. China; ^2^ Sinopec(Beijing) Research Institute of Chemical Industry Co., Ltd. National Engineering Research Center for Synthesis of Novel Rubber and Plastic Materials P. R. China; ^3^ School of Chemical Engineering and Technology Xi'an Jiaotong University Xi'an Shaanxi P. R. China

**Keywords:** mechanical properties, molecular dynamics simulations, physics‐embedded neural network, solution‐polymerized styrene‐butadiene rubber, transfer learning

## Abstract

Designing and developing high‐performance polymeric materials with machine learning has become a major trend in materials science. However, conventional data‐driven neural network models often suffer from the “black‐box” problem, lacking physical constraints and interpretability, and struggle to achieve accurate predictions with limited experimental data. In this work, a Physics‐Embedded Neural Network (PENN) is proposed, which incorporates the Yeoh hyperelastic constitutive model into the network architecture to embed physical laws directly into the learning process, thereby improving physical plausibility and interpretability. The model was first pre‐trained on large‐scale molecular dynamics (MD) simulation data to capture the intrinsic correlations between polymer structure and mechanical behavior. It was then fine‐tuned with a small amount of experimental data through a transfer learning strategy that calibrates the stress magnitude from high‐strain‐rate simulations to the experimental scale, effectively bridging the gap between simulation and experiment. Uncertainty quantification was further employed to validate the robustness of the predictions. Beyond accurate prediction, PENN enables performance‐driven inverse design by mapping target properties to candidate compositional regions, transforming predictive modeling into a practical tool for guiding polymer development.

## Graphical abstract

Traditional black‐box models for polymer mechanics rely solely on data and lack physical interpretability. This work presents a physics‐embedded neural network (PENN) that integrates constitutive equations into machine learning. The approach ensures reliable stress predictions, provides interpretable parameters, and enables performance‐driven, inverse design of polymeric materials.

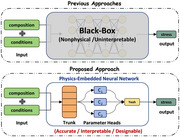

## 1 Introduction

Polymeric materials, owing to their outstanding mechanical properties, designability, and processability, are widely utilized in diverse fields such as automotive engineering, aerospace, energy, healthcare, and daily consumer products, making them indispensable functional materials in modern industrial systems [1, 2, 3, 4]. With the growing demands from emerging applications such as new‐energy transportation, intelligent manufacturing, and extreme service environments, stricter requirements are being placed on the mechanical performance of polymer, including thermal stability, fatigue resistance, and aging durability [5, 6, 7]. Achieving accurate prediction and rational design of macroscopic mechanical properties based on molecular structures and compositional formulations has thus become one of the central challenges in the development of high‐performance polymeric materials [8, 9, 10, 11].

Molecular dynamics (MD) simulations, as an advanced tool capable of characterizing structure‐property relationships at the molecular scale, have been extensively applied in polymer and rubber research [12, 13]. Compared with conventional experimental methods, MD simulations not only provide efficiency and controllability but also offer unique insights into microscopic mechanisms such as molecular interactions and strain responses. Furthermore, MD enables rapid evaluation of different molecular architectures, demonstrating distinct advantages in exploring structure‐property relationships and validating theoretical models [14, 15, 16]. However, due to limitations in computational resources and algorithmic efficiency, MD simulations are typically carried out at strain rates far higher than those encountered in real experiments. This mismatch from practical conditions restricts MD results mainly to qualitative analyses, thereby limiting their direct application to engineering optimization and industrial practice [17, 18].

The rapid development of machine learning (ML) has opened new avenues for accelerating formulation exploration and property prediction in materials science [19, 20, 21, 22, 23, 24]. ML models can efficiently capture complex and nonlinear relationships between molecular structures and macroscopic performance, significantly reducing reliance on costly experiments and large‐scale simulations. Recent researches have demonstrated that algorithms such as neural networks, gradient boosting trees, convolutional networks, and recurrent networks perform effectively in polymer property prediction [25, 26, 27, 28]. Nevertheless, conventional “black‐box” models are highly data‐driven, lacking explicit physical constraints and often yielding predictions inconsistent with fundamental mechanical laws. Moreover, the limited interpretability of these models hinders mechanism‐based formulation control and rational material design.

To address these limitations, integrating physical priors with ML has emerged as a promising research direction. Physics‐embedded strategies incorporate constraints such as conservation laws, boundary conditions, and energy dissipation into the model architecture or loss function, thereby guiding the learning process with physical laws [29, 30, 31, 32]. This approach not only improves predictive accuracy but also enhances extrapolation capability and interpretability [33, 34]. In the context of rubber materials, constitutive models serve as essential theoretical foundations for describing mechanical behavior. Embedding such constitutive relationships into ML frameworks offers a novel pathway to achieve predictions that are not only accurate but also physically structured and interpretable [35].

**FIGURE 1:**
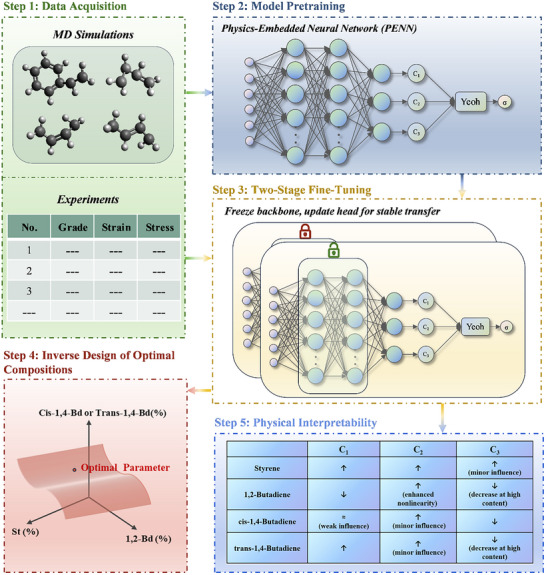
Workflow of the proposed physics‐embedded neural network. Step 1: Molecular dynamics simulations of SSBR systems with varying styrene, 1,2‐butadiene, cis‐1,4‐butadiene, and trans‐1,4‐butadiene contents under different applied conditions, producing stress–strain curves. Step 2: Pretraining of PENN using MD data with embedded Yeoh constitutive equations. Step 3: Two‐stage fine‐tuning with experimental data, including parameter‐head calibration and gradual unfreezing of the backbone. Step 4: Mapping of network outputs to constitutive parameters to enable physical interpretability. Step 5: Virtual experimentation across 7041 compositional combinations and inverse design through threshold‐based screening of candidate structural compositions.

To address these challenges, a Physics‐Embedded Neural Network (PENN) is proposed in this work. This approach integrates the Yeoh hyperelastic constitutive model into the neural network framework as a physically structured constraint, enabling the model not only to learn input‐output relationships but also to predict stress responses through a constitutive form that enhances model interpretability during training. Rather than serving as a complete constitutive theory, the embedded Yeoh formulation provides a stable and interpretable functional structure that helps reduce nonphysical predictions commonly encountered in purely data‐driven models. Specifically, the model is first pre‐trained on a large dataset generated from MD simulations to capture the correlations between stress, the molar fractions of four structural units, and strain rate. It is then fine‐tuned in two stages using a limited amount of experimental data to progressively calibrate the output scale and mitigate the distribution discrepancy between simulation and experiment. The incorporation of the constitutive model substantially enhances the interpretability of the network, moving beyond the “black‐box” state of purely data‐driven approaches and achieving high‐accuracy predictions. Furthermore, the PENN model enables systematic exploration of the compositional space and visualization of performance landscapes, providing a new inverse design strategy whereby candidate structural compositions can be screened according to target mechanical properties. This establishes an efficient “property‐to‐structure” pathway and offers a new strategy for interpretable prediction and intelligent design of polymeric materials. The overall workflow is illustrated in Figure 1. It should be noted that, under classical hyperelastic assumptions, strain rate is not treated as a constitutive variable. Its inclusion here is motivated by the need to decouple compositional effects from systematic differences between MD simulations and experimental testing conditions. If a single strain rate were used for each compositional configuration, the strain‐rate variable would effectively be fixed, leading the model to incorrectly attribute the elevated stress levels observed in simulations to material composition alone. By introducing multiple strain rates for the same structural composition, the model is able to distinguish variations arising from changes in structural unit content from those arising from differences in loading conditions. Accordingly, strain rate is treated in this work not as a viscoelastic variable, but as a conditioning variable that facilitates the alignment of mechanical response distributions between simulation and experiment.

## 2 Results and Discussion

### 2.1 Model Pretraining and Interpretability

**FIGURE 2:**
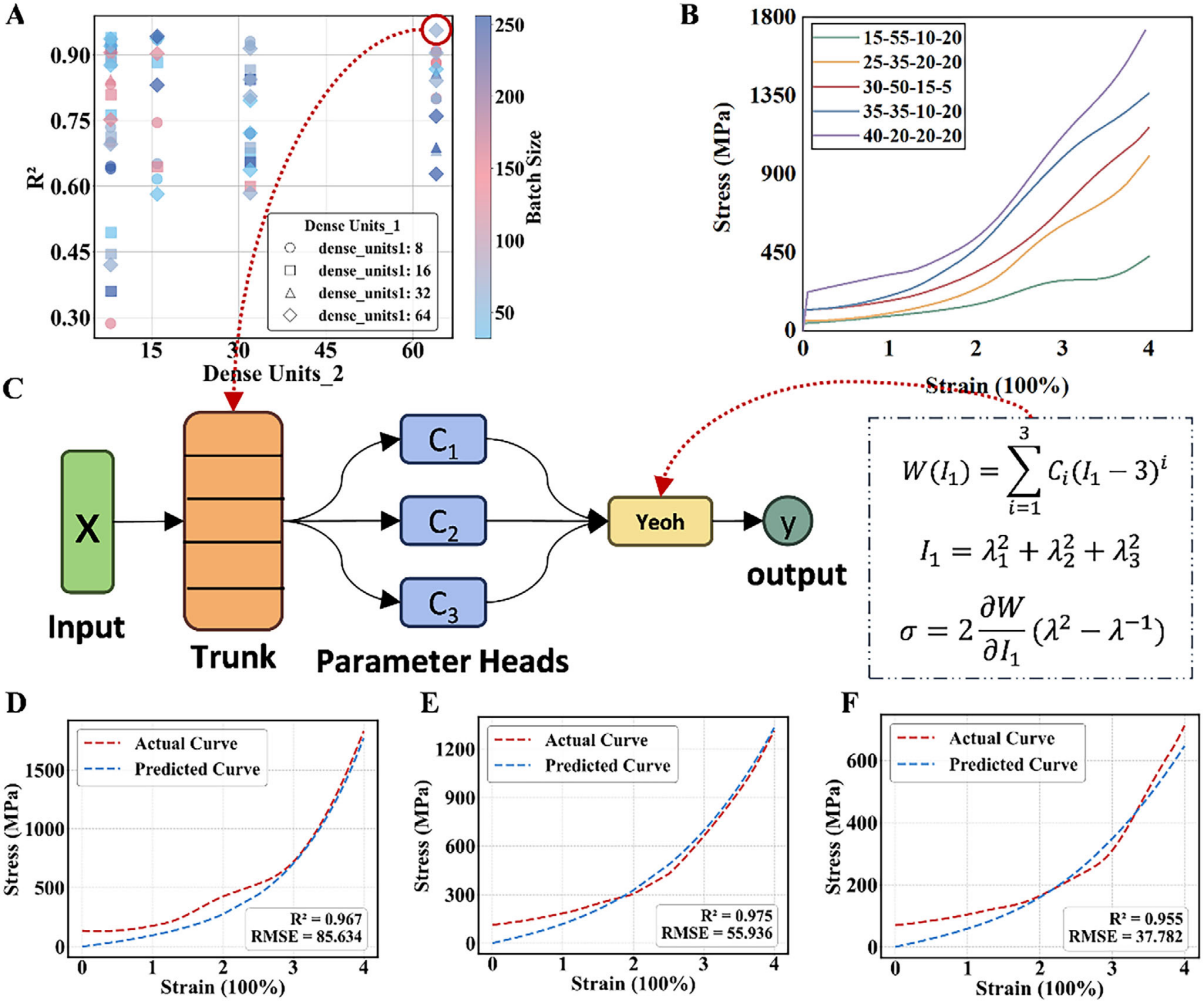
Model pretraining with MD simulation data. (A) Hyperparameter search using Bayesian optimization to determine network architecture. (B) Representative stress–strain curves generated by MD simulations across different strain rates. (C) Framework of PENN with embedded Yeoh equation for physically consistent stress computation. (D–F) Comparison between PENN predictions and MD curves under distinct compositions, showing excellent agreement with R^2^ > 0.95.

Solution‐polymerized styrene‐butadiene rubber (SSBR) was selected as a representative system. MD simulations were employed to construct 20 molecular models with varying compositions, encompassing four structural units: styrene, 1,2‐butadiene, cis‐1,4‐butadiene, and trans‐1,4‐butadiene. Each system was subjected to uniaxial tensile loading at five distinct strain rates, generating a total of 100 complete stress–strain curves. Details of the simulation procedures are provided in Section S1. These curves span a broad strain range and capture nonlinear deformation behavior, thereby providing a comprehensive dataset that characterizes stress–strain responses across different structural compositions. Representative examples are illustrated in Figure 2B.

For model input features, in addition to the molar fractions of the four structural units, strain and strain rate were included. The compositional descriptors encode differences in molecular structure, whereas strain is introduced to represent the deformation state. Strain rate is incorporated to explicitly distinguish the disparate loading time scales associated with MD simulations and experimental measurements, rather than to model intrinsic rate‐dependent material behavior. This feature design allows the model, during pretraining, to learn structure‐stress relationships while accounting for systematic shifts in stress levels arising from differences in loading conditions. As a result, the model is better conditioned for subsequent fine‐tuning with limited experimental data and for achieving reliable cross‐scale transfer between simulation and experiment.

To determine an appropriate network architecture, Bayesian optimization was performed to systematically search across hyperparameters such as hidden layer size and batch size (Figure 2A). Unlike conventional grid search or random search, Bayesian optimization efficiently explores the high‐dimensional parameter space by constructing a surrogate model that captures the relationship between hyperparameters and validation performance, thereby converging more rapidly to the optimal region [36, 37, 38]. In this work, the Bayesian optimizer operates on continuous auxiliary variables, which are subsequently discretized prior to model training. Specifically, the sampled continuous values are projected onto integers and mapped to powers of two, resulting in discrete candidate sets for the network width (hidden units ∈ {8, 16, 32, 64, 128}) and batch size (batch size ∈ {32, 64, 128, 256, 512}). This strategy enables efficient exploration using a continuous surrogate model while restricting the actual training to a practically relevant and computationally stable discrete hyperparameter set. The results showed that larger model capacity helps capture complex nonlinear relationships, but excessive parameters lead to unstable training. Ultimately, a two‐hidden‐layer architecture was adopted, each with 64 neurons and ReLU activation, balancing nonlinear representation and computational efficiency. To mitigate overfitting and enhance generalization, an L2 regularization term was added to the loss function. The Adam optimizer was employed with an initial learning rate of 1 × 10^−5^, and a gradient clipping schedule was applied: if validation loss plateaued for 5 consecutive epochs, the learning rate was halved, with a minimum of 1 × 10^−7^. An early‐stopping criterion was also used, terminating training if no improvement was observed for 20 epochs, after which the best‐performing weights were restored. These strategies effectively suppressed overfitting and stabilized training. The final network achieved an accurate prediction of complex stress responses while preserving physical consistency.

Distinct from purely data‐driven black‐box models, the proposed PENN incorporates three parallel output heads corresponding to the Yeoh constitutive parameters {C_1_, C_2_, C_3_}, with Softplus activation to ensure parameter non‐negativity [39]. By mapping latent features directly into constitutive parameters and substituting them into the stress formulation, the model enforces strict compliance with nonlinear elasticity theory:

(1) $$W\left(I_{1}\right)=\sum_{i=1}^{3}C_{i}{\left(I_{1}-3\right)}^{i}$$

where $I_{1}=\lambda_{1}^{2}+\lambda_{2}^{2}+\lambda_{3}^{2}$ is the first invariant. For uniaxial tension, the principal stretches satisfy:

(2) $$\lambda_{1}=\lambda ;\;\lambda_{2}=\lambda_{3}=\lambda^{-\frac{1}{2}}$$

Thus, *I*_1_ = λ^2^ + 2λ^−1^, and the stress $\sigma =2\frac{\partial W}{\partial I_{1}}\left(\lambda^{2}-\lambda^{-1}\right)$ can be expressed as:

(3) $$\begin{array}{ccc} \sigma & = & 2\left(\lambda^{2}-\lambda^{-1}\right)\\ & & \times \left[C_{1}+2C_{2}\left(\lambda^{2}+2\lambda^{-1}-3\right)+3C_{1}{\left(\lambda^{2}+2\lambda^{-1}-3\right)}^{2}\right] \end{array}$$

This embedding strategy ensures that the model no longer performs empirical mapping from inputs to outputs in a purely data‐driven manner. Instead, it predicts stress responses through strain energy functions grounded in continuum mechanics, thereby preventing unphysical results (e.g., negative modulus or non‐monotonic stress curves) and substantially improving interpretability.

To evaluate predictive accuracy, model outputs were compared with MD simulation data for three test curves (Figure 2D–F). Across different compositions, the predicted curves closely matched the simulations, with R^2^ values exceeding 0.95 and RMSE within a reasonable range. This demonstrates that the physics‐embedded strategy significantly enhances the expressive capability of the model during pretraining and provides a solid foundation for fine‐tuning with scarce experimental data.

**FIGURE 3:**
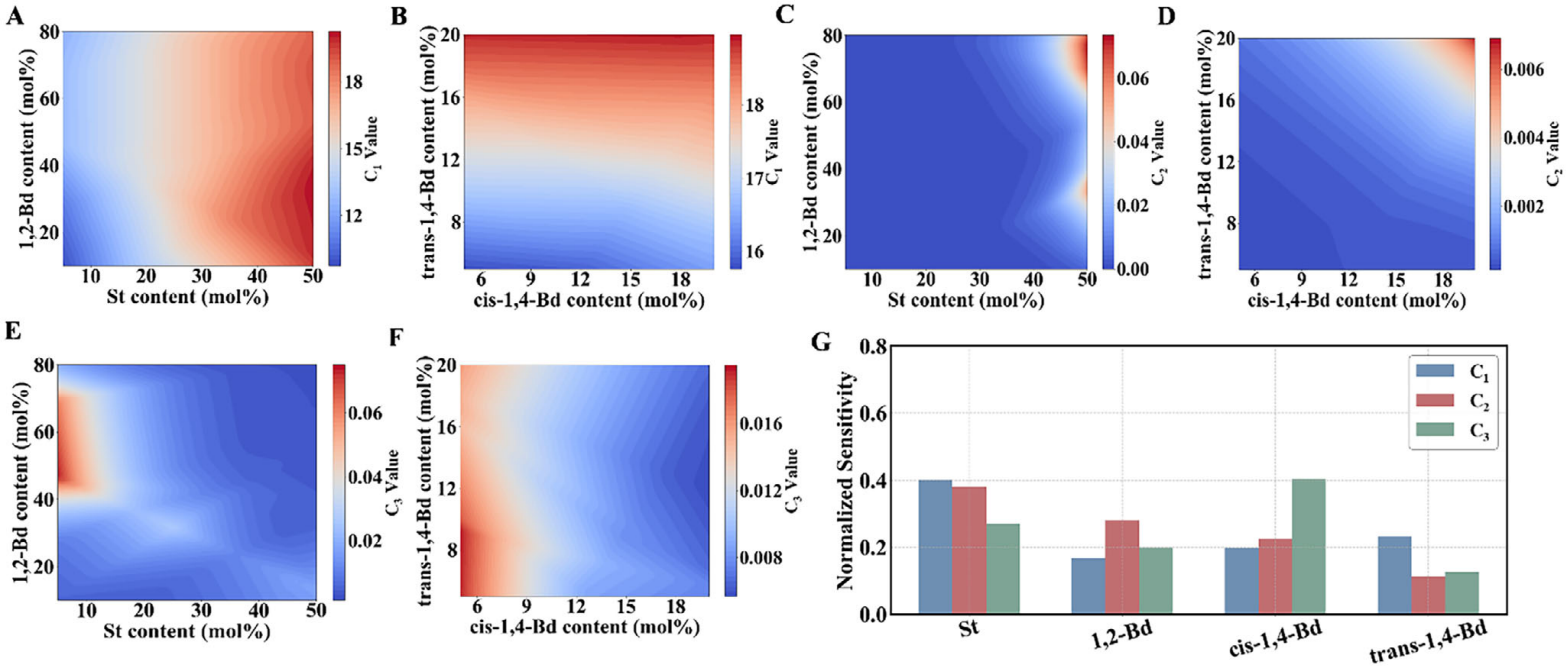
Dependence of Yeoh constitutive parameters on structural unit contents. (A,B) Variation of C_1_ with styrene, 1,2‐butadiene, cis‐1,4‐butadiene, and trans‐1,4‐butadiene contents. (C,D) Variation of with C_2_ the four structural units. (E,F) Variation of C_3_ with the four structural units. (G) Normalized sensitivity of C_1_, C_2_, and C_3_ with respect to individual structural unit contents.

Furthermore, the incorporation of physical information not only improved predictive accuracy but also enhanced interpretability. Figure 3 illustrates the variation of the effective Yeoh parameters {C_1_, C_2_, C_3_} with the contents of the four structural units. It should be emphasized that these parameters are effective quantities inferred by the model under specified loading conditions, rather than intrinsic material constants in the classical constitutive sense. As such, their variations reflect how changes in molecular composition are mapped by the model onto different deformation regimes of the stress–strain response.

In Figure 3A–F, 2D heat maps are constructed by varying pairs of structural unit contents while fixing the remaining inputs at their mean values, enabling visualization of compositional trends in the effective parameters. To further enable quantitative comparison of the relative influence of individual structural units, a sensitivity analysis was performed. Specifically, each structural unit content was perturbed by a small amount (1 mol%) while keeping the other inputs unchanged, and the resulting changes in C_1_, C_2_, and C_3_ were evaluated and normalized across the dataset, as summarized in Figure 3G.

The combined heat map and sensitivity analyses reveal distinct roles of different structural units across deformation regimes. C_1_, which dominates the small‐strain response, shows strong dependence on styrene and trans‐1,4‐butadiene contents, consistent with enhanced initial stiffness associated with rigid and more regular chain structures, while increasing 1,2‐butadiene content leads to a reduction in C_1_, reflecting increased chain flexibility. In contrast, C_2_ remains relatively small overall, indicating weak strain‐hardening behavior at intermediate strains, but exhibits sensitivity to variations in multiple structural units, suggesting that intermediate‐strain responses arise from the combined effects of different compositional features. For the higher‐order parameter C_3_, which primarily affects large‐deformation behavior, sensitivity is dominated by cis‐1,4‐butadiene content, consistent with the role of chain flexibility and conformational freedom in governing nonlinear deformation. Collectively, these results demonstrate that the PENN framework establishes a consistent and interpretable mapping between molecular composition and the effective parameters controlling different deformation regimes, thereby providing improved physical insight compared to purely data‐driven models.

### 2.2 TwoStage Fine‐Tuning

**FIGURE 4:**
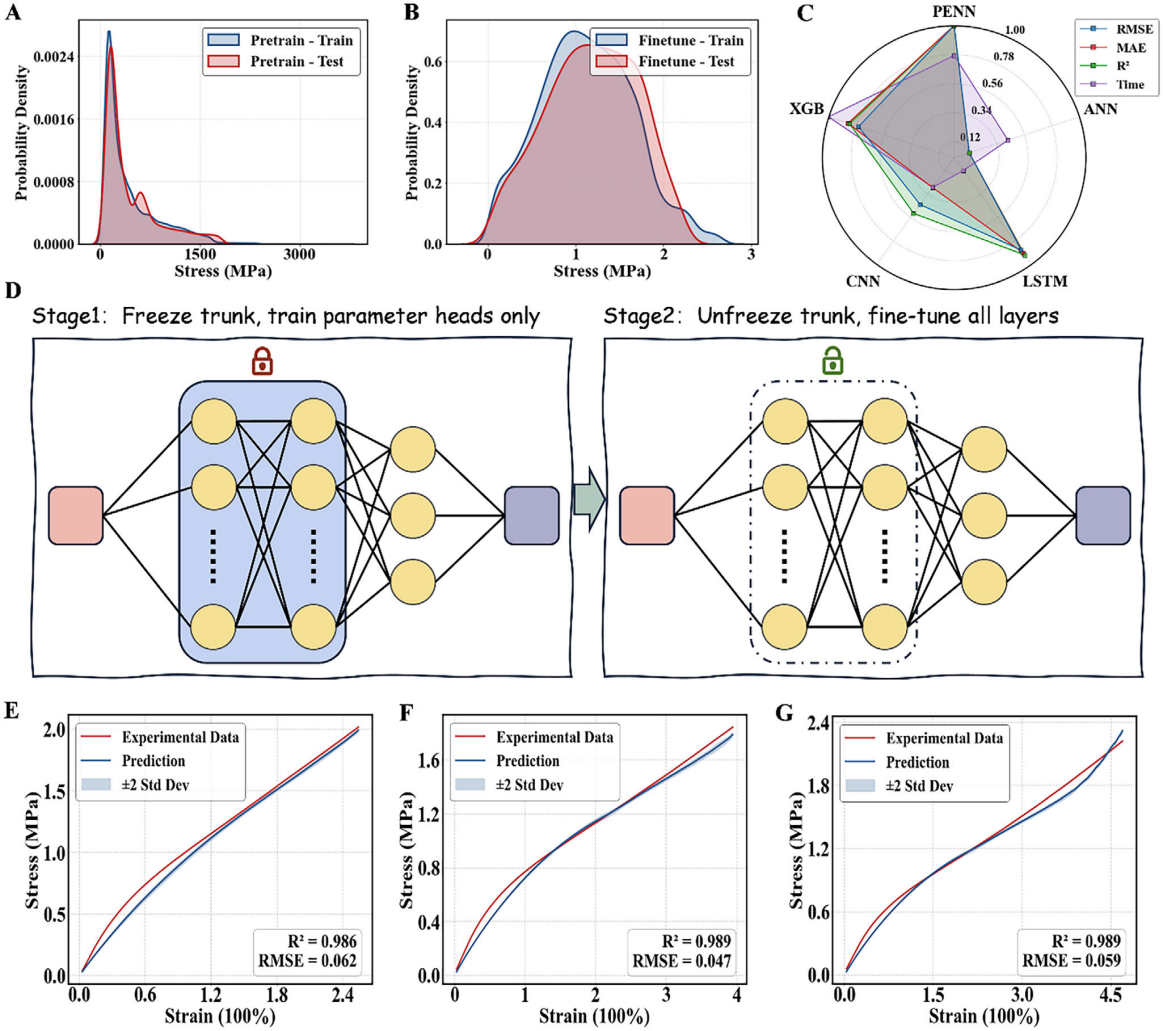
Applications of PENN in experimental calibration, model comparison, and inverse design. (A,B) Discrepancies between PENN predictions and experimental stress prior to fine‐tuning. (C) Radar chart comparing ANN, LSTM, CNN, XGBoost, and PENN in terms of prediction accuracy (RMSE, MAE, and R^2^) and computational cost (training time). (D) Schematic illustration of the two‐stage fine‐tuning process. (E–G) Predictions for the test set after two‐stage fine‐tuning, including uncertainty quantification; shaded regions indicate ±2 standard deviations, reflecting the model's prediction confidence.

Although the pretraining stage significantly improved the accuracy and interpretability of the model, new challenges arise under real experimental conditions, particularly in small‐sample scenarios where model performance is severely constrained. Directly training a deep model on limited experimental data often encounters two major issues: first, a scale mismatch between predictions and actual measurements, manifested as significant numerical deviation (Figure 4A,B); and second, catastrophic forgetting, where training on new tasks disrupts the pretrained feature distribution and causes the model to forget the embedded physical knowledge [40, 41, 42, 43]. To address these issues, a two‐stage fine‐tuning strategy was employed, in which the degree of freedom of the model was gradually released to ensure stable convergence under small‐sample conditions [44].

Specifically, in Stage I, only the three parameter heads were fine‐tuned, while the earlier feature extraction layers were kept frozen. Since the backbone of the model captures the mapping from molecular structures to constitutive parameters, directly updating these parameters could cause severe perturbation to the learned feature distribution. By restricting training to the output heads in Stage I, the predicted parameter range could be rapidly calibrated to the scale of experimental data, avoiding the destruction of established physical representations caused by large‐scale parameter updates. Moreover, this step helped prevent overfitting under data‐scarce conditions, as the limited number of trainable parameters imposed a strong regularization effect.

On this basis, Stage II gradually unfroze the network for joint fine‐tuning, ensuring that the previously learned features would not be forgotten too quickly but gradually adapted to the new data distribution through slow gradient updates. This strategy demonstrated significant advantages in practice: it avoided the instability of end‐to‐end training, while simultaneously leveraging pretrained knowledge to enhance the physical generalization capacity of the model under limited data.

In practice, three commercially available SSBR grades were stretched at five different strain rates, yielding a total of 15 stress–strain curves. Detailed formulations and experimental procedures are provided in Section S2. Among these, 12 curves were used as the training set for fine‐tuning, while the remaining three served as a test set to evaluate the effectiveness of the fine‐tuning stage. In Stage I, the backbone network was frozen and only the parameter head was updated, allowing the simulated outputs to quickly adjust to the same magnitude as experimental results and ensuring a smooth transfer process. In Stage II, the entire network was unfrozen for global fine‐tuning, further refining the feature mappings and improving stress predictions across different structural compositions. The final predictions of the three test curves showed strong agreement with experimental values, as illustrated in Figure 4E–G, demonstrating the high predictive accuracy of the model.

Nevertheless, due to the limited experimental data, predictions from a single model may still be influenced by sampling variability and parameter initialization. To evaluate and enhance the robustness of the predictions, an uncertainty quantification approach was implemented. Specifically, multiple fine‐tuning runs were conducted starting from the same pretrained weights but using different random seeds, generating an ensemble of nearly independent models. The ensemble predictions were averaged, and the standard deviation was computed to form a confidence interval. The shaded regions in Figure 4E–G represent ±2 standard deviations, providing a direct visualization of the model's prediction uncertainty. The relatively narrow error bands and their close agreement with experimental results indicate that the model remains highly stable and reliable even with a limited number of experimental samples.

### 2.3 Model Comparison and Inverse Design

To further evaluate the advantages of the proposed approach, four widely used models—Artificial Neural Network (ANN), Long Short‐Term Memory Network (LSTM), Convolutional Neural Network (CNN), and Extreme Gradient Boosting (XGBoost)—were selected for horizontal comparison against PENN. For a fair and consistent comparison, all benchmark models were trained and evaluated using exactly the same input feature set as the PENN model. Therefore, the performance differences observed among the models do not stem from unequal access to input information, but rather reflect the impact of incorporating physically structured constraints in the network architecture. The baseline models serve as purely data‐driven references without embedded physical formulations, allowing the benefit of the physics‐embedded strategy to be assessed under identical information conditions. To provide an intuitive comparison of model performance across multiple evaluation metrics, a radar chart was constructed using three normalized indicators: RMSE, MAE, and R^2^. In addition, the total training time of each model was included as a complementary indicator to assess computational cost, enabling a joint evaluation of predictive accuracy and training efficiency. As RMSE and MAE are error‐based metrics for which smaller values indicate better performance, they were inversely normalized for visualization so that all indicators follow a consistent “higher‐is‐better” convention, whereas R^2^ was normalized directly, and the training time was inversely normalized accordingly(Figure 4C). The result shows that PENN consistently outperforms the other models in both RMSE and MAE, while also achieving the highest R^2^. Notably, despite its physically embedded architecture, PENN does not incur a prohibitive computational burden; its overall training time is comparable to or even lower than that of several deep learning baselines, such as ANN, CNN, and LSTM, demonstrating a favorable balance between accuracy and efficiency. By contrast, the ANN, which lacks physical constraints, relies purely on nonlinear fitting and thus often generates predictions that deviate from underlying material mechanisms, leading to high variability and poor generalization as well as extended training time due to inefficient exploration of the parameter space. LSTM, although advantageous for capturing sequential dependencies, underperforms in this work because the nonlinear relationships driven by compositional features dominate the predictive task, while its recurrent structure introduces additional computational overhead during training. XGBoost, based on piecewise tree ensembles, provides rapid fitting of nonlinear interactions but tends to produce segmented and less smooth responses, inconsistent with the continuity of stress–strain curves, even though it benefits from relatively low training cost. CNN, while effective at local feature extraction, is inherently limited in tasks requiring global physical consistency, resulting in weaker overall performance and comparatively high training time due to deeper network depth. Taken together, these comparisons highlight that PENN, by embedding physical information, not only improves predictive accuracy but also enhances stability and interpretability, while maintaining competitive training efficiency, delivering a more reliable modeling framework than purely data‐driven methods.

**FIGURE 5:**
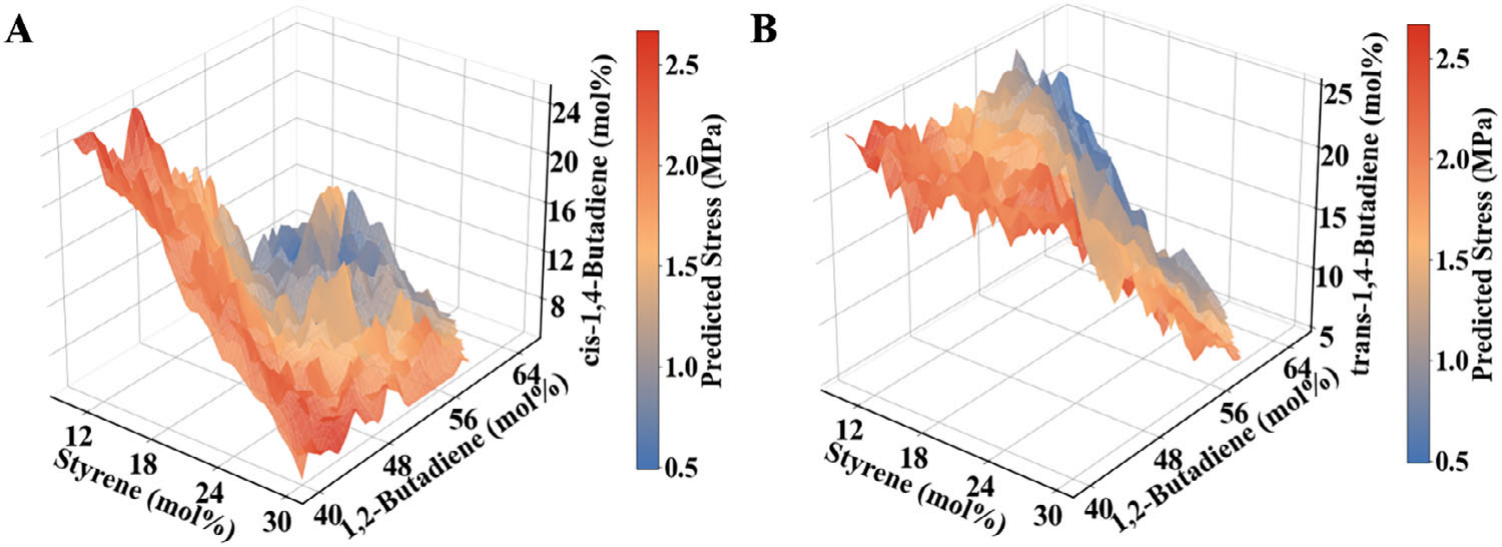
Predicted stress at 300% fixed strain for 7041 SSBR compositional combinations. (A) cis‐1,4‐butadiene and (B) trans‐1,4‐butadiene are used as the *Z*‐axis in the 3D heat maps, illustrating how composition affects mechanical performance.

Having established the reliability of the predictive model, PENN was further employed as a “virtual experimentation platform” to systematically explore 7041 candidate compositional combinations within practically relevant ranges. These combinations were constructed by varying the molar fractions of the four structural units within predefined ranges using a fixed increment of 1 mol%, while retaining only those compositions whose total molar fraction sums to 100 mol%. The resulting compositional space, therefore, consists of physically valid and practically relevant SSBR formulations. For each combination, the stress response at a fixed strain of 300% was computed, and the corresponding performance heat maps were constructed (Figure 5A,B). These maps provide a clear visualization of how variations in composition influence mechanical performance, while enabling a systematic exploration of the compositional space that would be difficult to achieve through experiments alone. To further assess the reliability of these large‐scale inverse predictions, uncertainty analysis and additional experimental validation were conducted, as detailed in the Supporting Information.

On this basis, a property‐driven inverse design strategy was developed. Rather than treating the design process as a black‐box optimization or purely mathematical inversion, the approach systematically explores the feasible composition space and applies target‐based thresholds to identify candidate structural compositions. This strategy does not yield a single solution but highlights a set of possible regions that satisfy the performance criteria, allowing flexibility for further refinement through targeted experiments or simulations. In this way, the predictive model is translated into a practical tool for performance‐oriented materials design.

## 3 Conclusion

In this research, a PENN was proposed using SSBR as a representative system. The approach overcomes the limitations of MD simulations in mechanical property prediction as well as the challenge of data scarcity in experiments, enabling efficient prediction of mechanical performance from the molecular scale to the mechanical performance level. By embedding a physically structured Yeoh hyperelastic formulation into the network architecture, the PENN constrains stress prediction through a stable constitutive form, thereby reducing physically implausible responses often produced by conventional black‐box models while improving predictive accuracy and interpretability. During pretraining, the model learns transferable relationships between molecular composition and stress–strain responses from a large MD‐generated dataset. Under limited experimental data, the two‐stage fine‐tuning strategy effectively corrected scale deviations and mitigated catastrophic forgetting, while uncertainty quantification further reduced the influence of data scarcity and enhanced prediction reliability.

Comparative evaluations against ANN, LSTM, CNN, and XGBoost demonstrated that the PENN achieved superior performance across all key metrics. Building on this advantage, PENN was used as a “virtual experimentation platform” to systematically traverse common structural unit combinations and generate performance heat maps, providing a clear visualization of how composition modulates mechanical behavior and expanding the exploration space beyond what experiments can feasibly cover. Based on these results, performance‐driven inverse design was realized by screening candidate structural compositions that meet predefined performance thresholds, offering flexibility for further refinement through experiments or simulations and transforming predictive modeling into a practical tool for materials design.

Looking forward, the PENN framework represents an extensible modeling paradigm rather than a material‐specific solution. Its application to other polymer systems or more complex experimental conditions would require the selection of physical representations and constraints consistent with the dominant deformation mechanisms of the target material. In future work, the PENN framework may be extended to a broader range of polymer systems and more complex experimental scenarios by incorporating physical representations and constraints that better reflect the dominant deformation mechanisms of the target materials. In particular, when reliable descriptors are available, it would be of interest to enrich the current composition‐level inputs by including additional microstructural information, such as sequence distribution, chain topology, and crosslink density, which are known to influence rubber elasticity. Such extensions may broaden the applicability of the framework and further improve predictive performance and interpretability, while keeping the modeling assumptions and data requirements explicit. Furthermore, integration with active learning and generative modeling could enable more efficient, performance‐guided exploration of polymer design spaces, thereby facilitating a closed‐loop workflow that links prediction with targeted experimental validation.

## Conflicts of Interest

The authors declare no conflicts of interest.

## Supporting information




**Supporting File 1**: advs74095‐sup‐0001‐SuppMat.docx

## Data Availability

The data that support the findings of this study are available from the corresponding author upon reasonable request.
