# Supplementary material for: Physics‐Embedded Neural Network: A Novel Approach to Design Polymeric Materials
Source: Adv Sci (Weinh). 2026 Jan 28:e22475. Online ahead of print. doi: 10.1002/advs.202522475 (PMC13325832; doi:10.1002/advs.202522475)
Supplement: Supplementary file 1 — Supporting File 1: advs74095‐sup‐0001‐SuppMat.docx [file ADVS-9999-e22475-s001.docx]

**Supporting Information for**

**Physics-Embedded Neural Network: A Novel Approach to Design Polymeric Materials**

Siqi Zhan^1^, Hengheng Zhao^1^, Haotian Wang^1^, Zhanjie Liu^1^, Qian Li^1^, Weifeng Zhang^1^, Qingsong Zhao^2^, Liqun Zhang^1,3^, and Jun Liu^1^*

^1^ State Key Laboratory of Organic-Inorganic Composites, Beijing University of Chemical Technology, Beijing 100029, People’s Republic of China

^2^ Sinopec (Beijing) Research Institute of Chemical Industry Co., Ltd. National Engineering

Research Center for Synthesis of Novel Rubber and Plastic Materials

^3^School of Chemical Engineering and Technology, Xi’an Jiaotong University, Xi’an, Shaanxi, 710049, People’s Republic of China

*Correspondence: [liujun@mail.buct.edu.cn](mailto:liujun@mail.buct.edu.cn)

**Section1. Molecular Dynamics Simulations and Stress-Strain Responses of SSBR Systems**

To enable the neural network to effectively learn the relationships between molecular composition, applied conditions, and stress responses, molecular dynamics (MD) simulations were performed as the primary source of pretraining data. These simulations provided a systematic dataset spanning a broad range of structural unit combinations and strain rates, ensuring that the model could capture diverse deformation behaviors beyond the limited scope of experimental measurements.

The molecular structures of solution-polymerized styrene-butadiene rubber (SSBR) were generated using Materials Studio, where random copolymerization was adopted to construct chains comprising four structural units: styrene, 1,2-butadiene, cis-1,4-butadiene, and trans-1,4-butadiene. The molar ratios of these units were systematically varied to generate 20 distinct SSBR systems, each consisting of 20 polymer chains. Detailed compositions are summarized in Table S1.

After construction, each system underwent equilibration within the NPT ensemble, employing the Velocity-Verlet algorithm with a 1 fs timestep. Temperature conditions were gradually adjusted through annealing cycles (600 K to 298 K) to achieve well-relaxed chain conformations. To mimic vulcanization, sulfur atoms were incorporated into the models, randomly crosslinking double bonds between adjacent chains. A sulfur dosage of 2 phr was consistently applied across all systems. Post-crosslinking, extended equilibration and annealing procedures ensured structural stability, with system density and non-bonded interactions converging to equilibrium values.

**Table S1** Structural unit compositions of SSBR from MD simulations (mol%)

| No. | Styrene Content | 1,2-Butadiene Content | Cis-1,4-Butadiene Content | Trans-1,4-Butadiene Content |
| --- | --- | --- | --- | --- |
| 1 | 5 | 55 | 20 | 20 |
| 2 | 10 | 50 | 20 | 20 |
| 3 | 25 | 35 | 20 | 20 |
| 4 | 40 | 20 | 20 | 20 |
| 5 | 50 | 10 | 20 | 20 |
| 6 | 5 | 65 | 10 | 20 |
| 7 | 15 | 55 | 10 | 20 |
| 8 | 25 | 45 | 10 | 20 |
| 9 | 35 | 35 | 10 | 20 |
| 10 | 45 | 25 | 10 | 20 |
| 11 | 10 | 70 | 15 | 5 |
| 12 | 20 | 60 | 15 | 5 |
| 13 | 30 | 50 | 15 | 5 |
| 14 | 40 | 40 | 15 | 5 |
| 15 | 50 | 30 | 15 | 5 |
| 16 | 10 | 80 | 5 | 5 |
| 17 | 20 | 70 | 5 | 5 |
| 18 | 30 | 60 | 5 | 5 |
| 19 | 40 | 50 | 5 | 5 |
| 20 | 50 | 40 | 5 | 5 |

Mechanical testing was then simulated through uniaxial tensile deformation under constant volume. The simulation box was elongated along the Z direction while the X and Y dimensions contracted proportionally to preserve volume. Stress was computed using the deviatoric stress tensor with a Poisson’s ratio of *μ* = 0.5:

$\sigma=(1+\mu)\left(\frac{1}{3}\sum_{i} P_{ii}-P_{zz}\right)$ (1)

Five engineering strain rates were considered—$\text{1×}\text{10}^{\text{12}}\text{/s}$, $\text{5×}\text{10}^{\text{11}}\text{/s}$, $\text{1×}\text{10}^{\text{11}}\text{/s}$, $\text{5×}\text{10}^{\text{10}}\text{/s}$, and $\text{1×}\text{10}^{\text{10}}\text{/s}$. For each of the 20 systems, tensile simulations were performed at all five rates, resulting in a total of 100 stress-strain datasets. The complete set of stress-strain curves is presented in Figure S1-S20, organized according to the compositional variables outlined above. Each figure displays five curves corresponding to different strain rates, distinguished by color coding and explicitly labeled in the legend. This arrangement allows straightforward comparison of rate-dependent mechanical responses for each SSBR microstructural configuration.


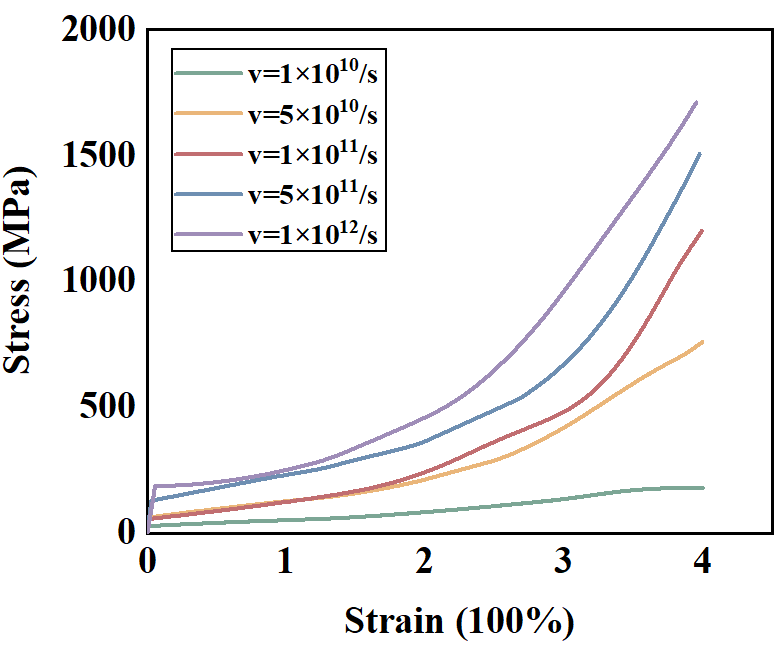


**Figure S1** Stress-Strain Curves corresponding to the composition 5-55-20-20.

**
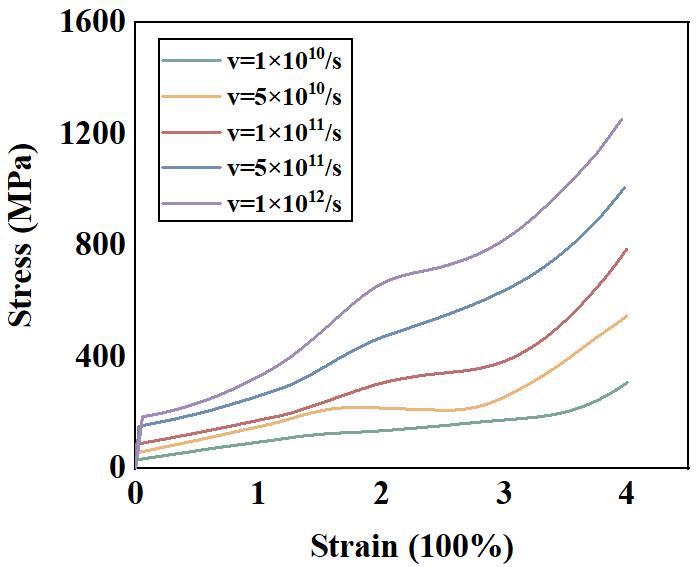
**

**Figure S2** Stress-Strain Curves corresponding to the composition 10-50-20-20.

**
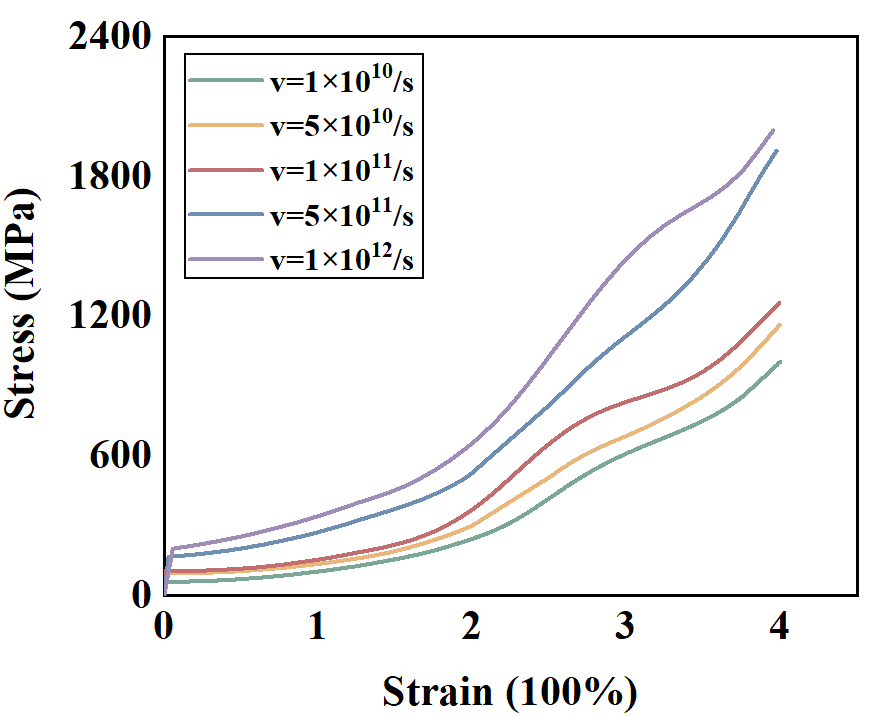
**

**Figure S3** Stress-Strain Curves corresponding to the composition 25-35-20-20.

**
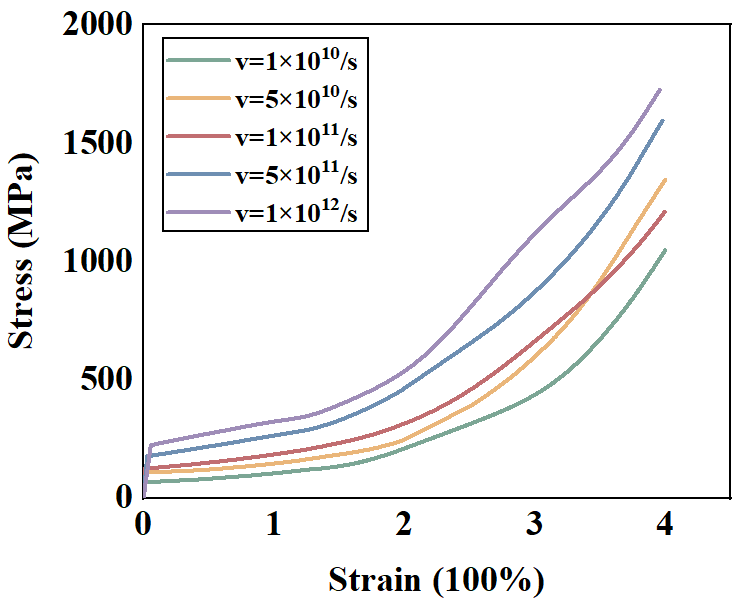
**

**Figure S4** Stress-Strain Curves corresponding to the composition 40-20-20-20.

**
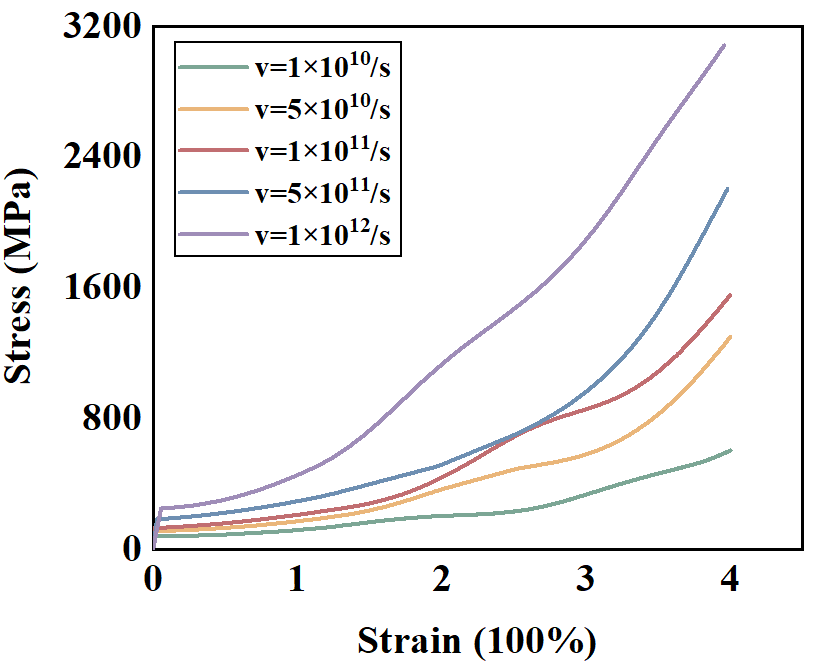
**

**Figure S5** Stress-Strain Curves corresponding to the composition 50-10-20-20.

**
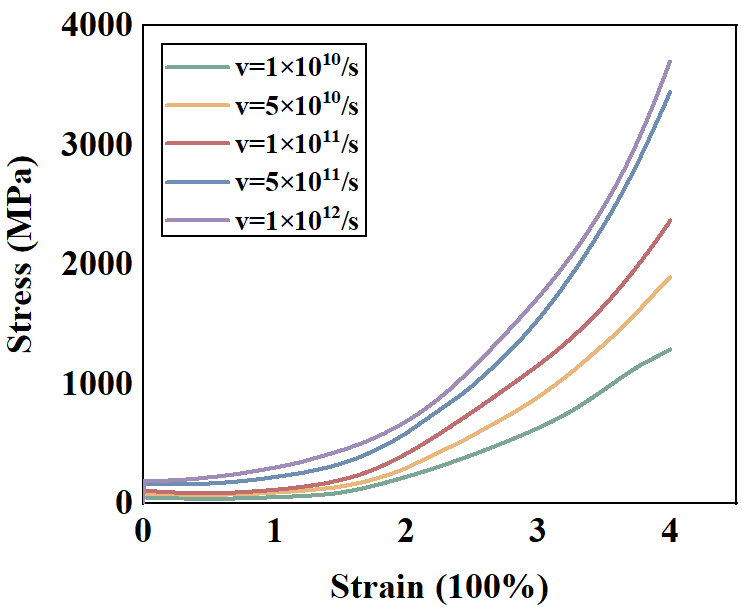
**

**Figure S6** Stress-Strain Curves corresponding to the composition 5-65-10-20.

**
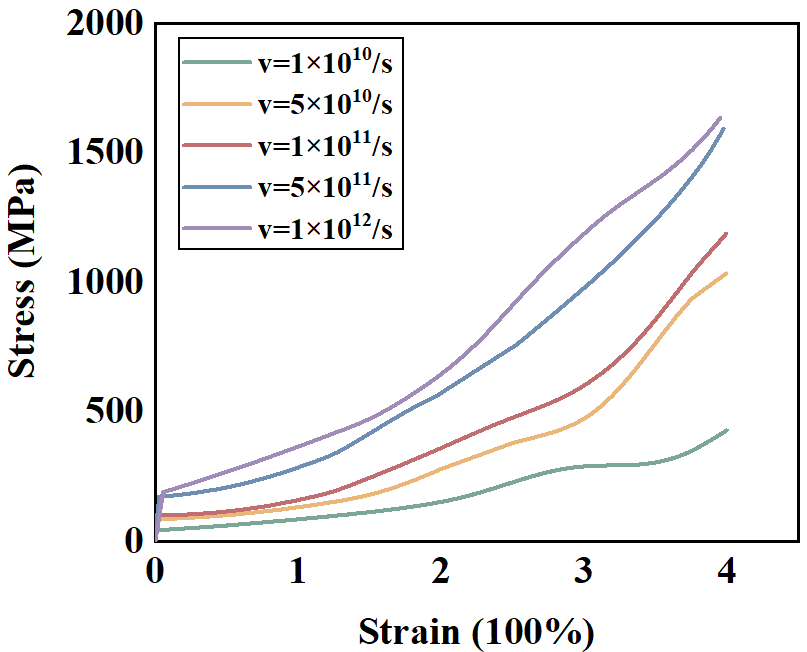
**

**Figure S7** Stress-Strain Curves corresponding to the composition 15-55-10-20.

**
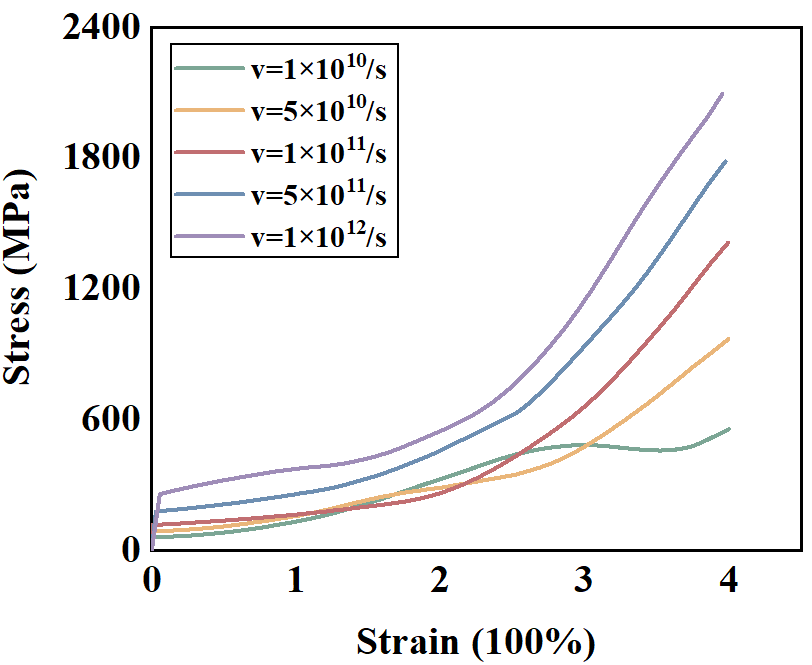
**

**Figure S8** Stress-Strain Curves corresponding to the composition 25-45-10-20.

**
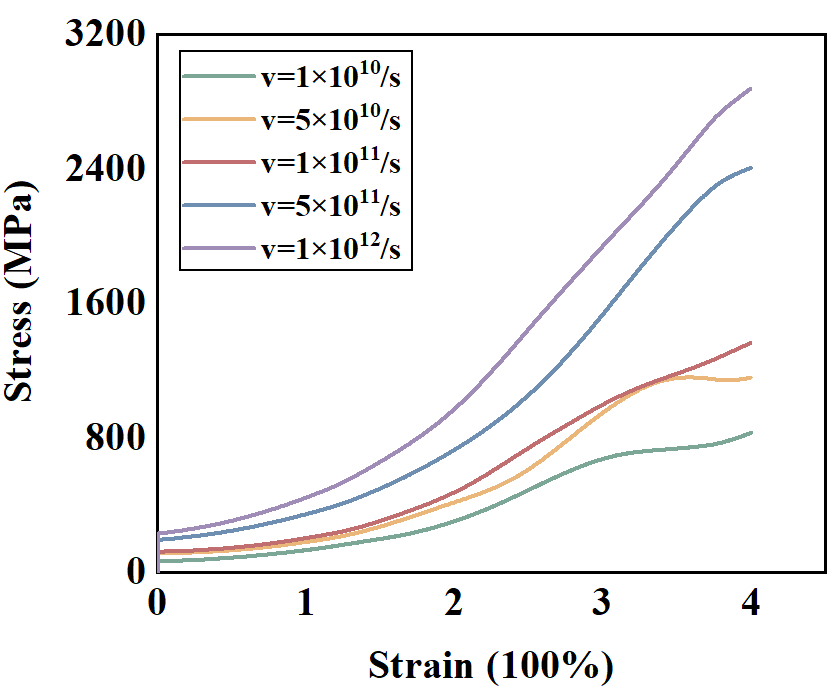
**

**Figure S9** Stress-Strain Curves corresponding to the composition 35-35-10-20.

**
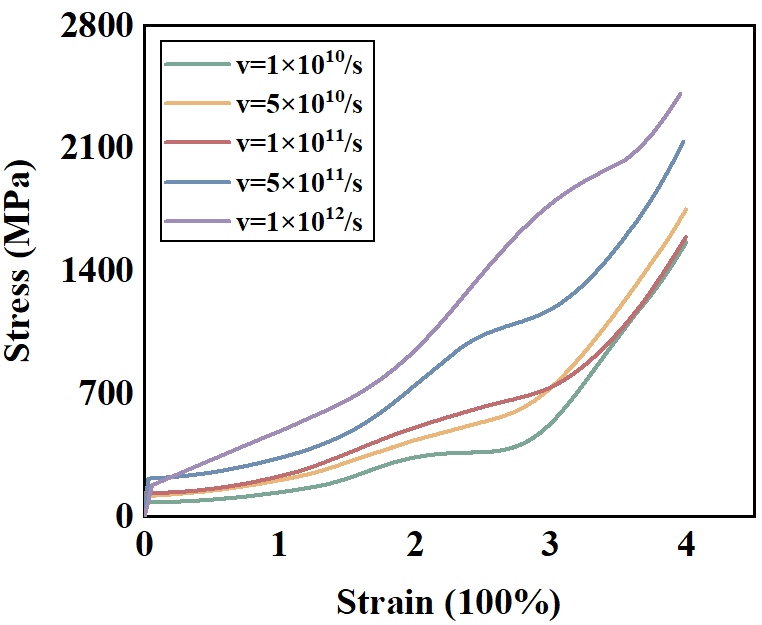
**

**Figure S10** Stress-Strain Curves corresponding to the composition 45-25-10-20.

**
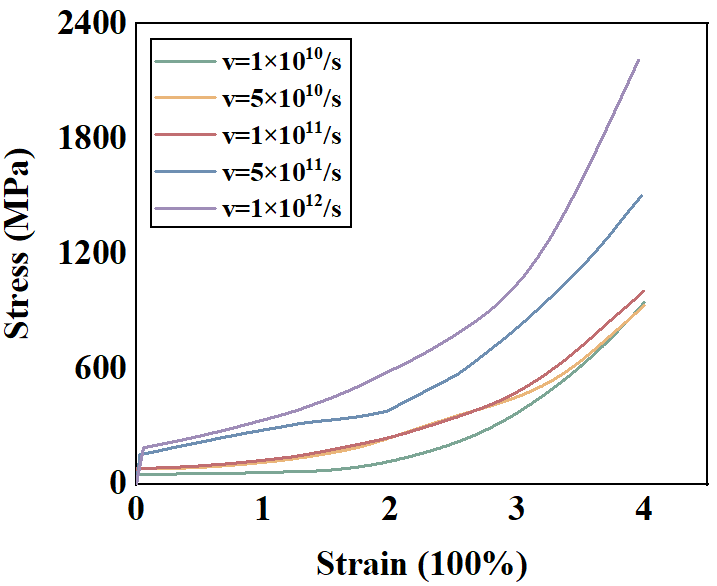
**

**Figure S11** Stress-Strain Curves corresponding to the composition 10-70-15-5.

**
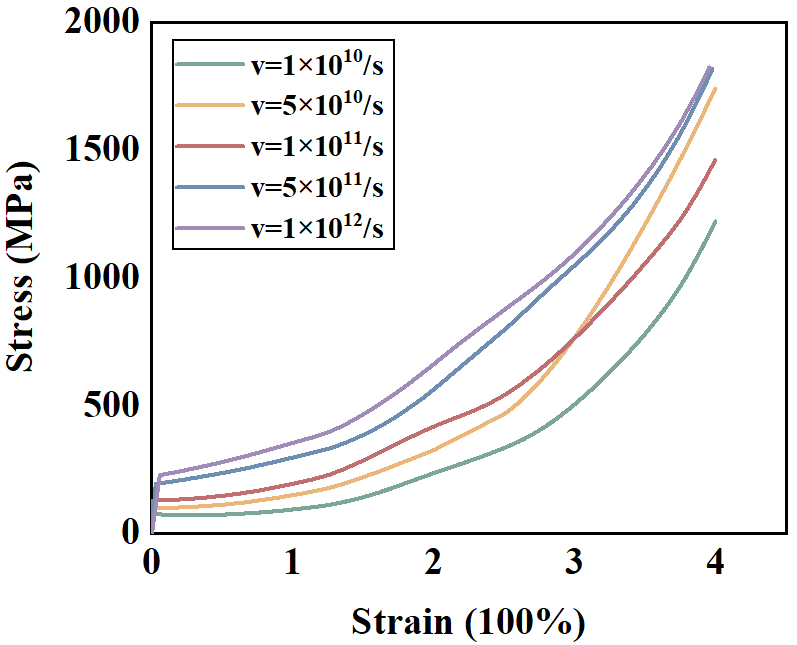
**

**Figure S12** Stress-Strain Curves corresponding to the composition 20-60-15-5.

**
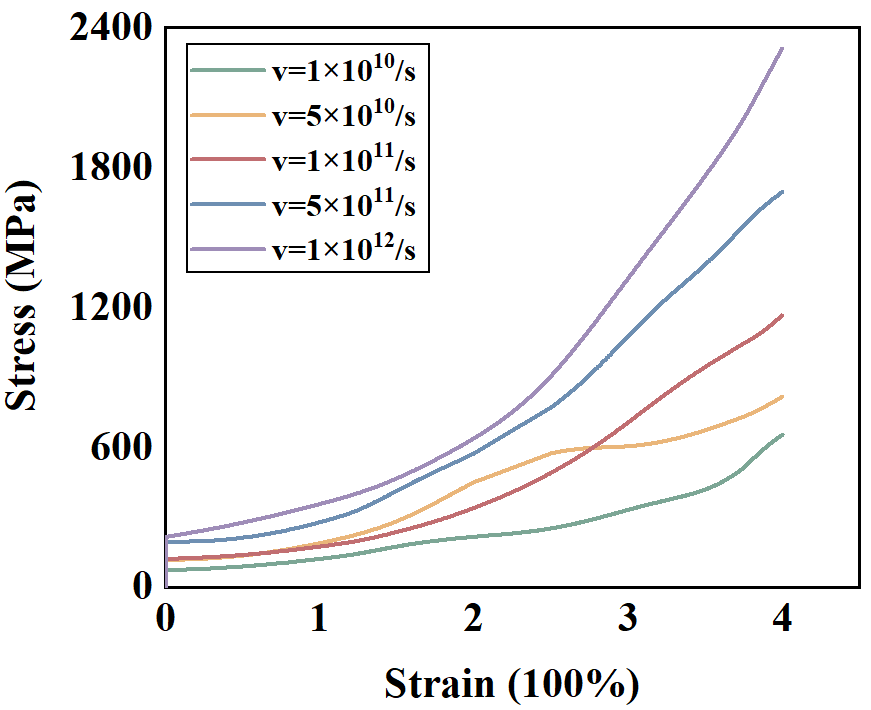
**

**Figure S13** Stress-Strain Curves corresponding to the composition 30-50-15-5.

**
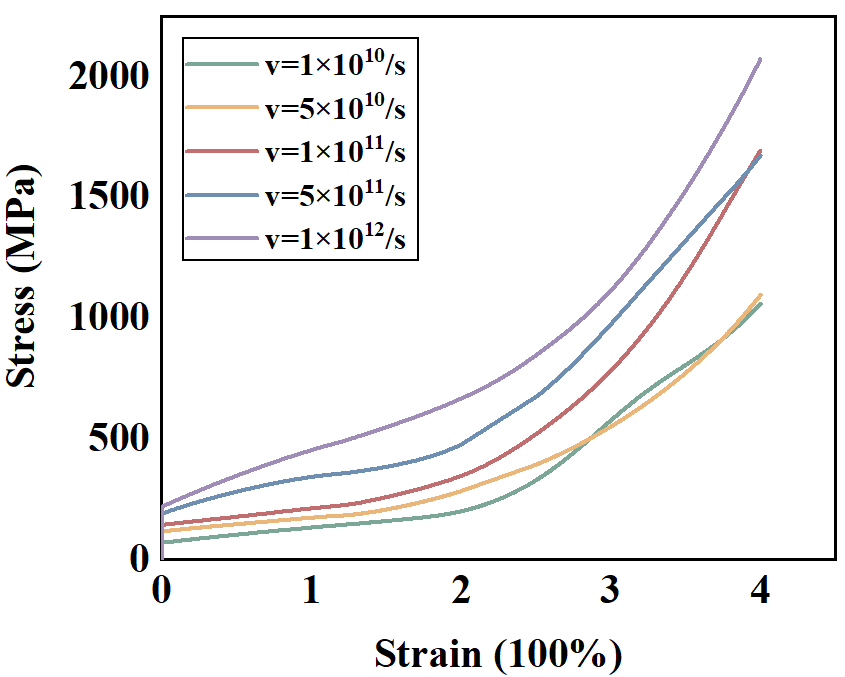
**

**Figure S14** Stress-Strain Curves corresponding to the composition 40-40-15-5.

**
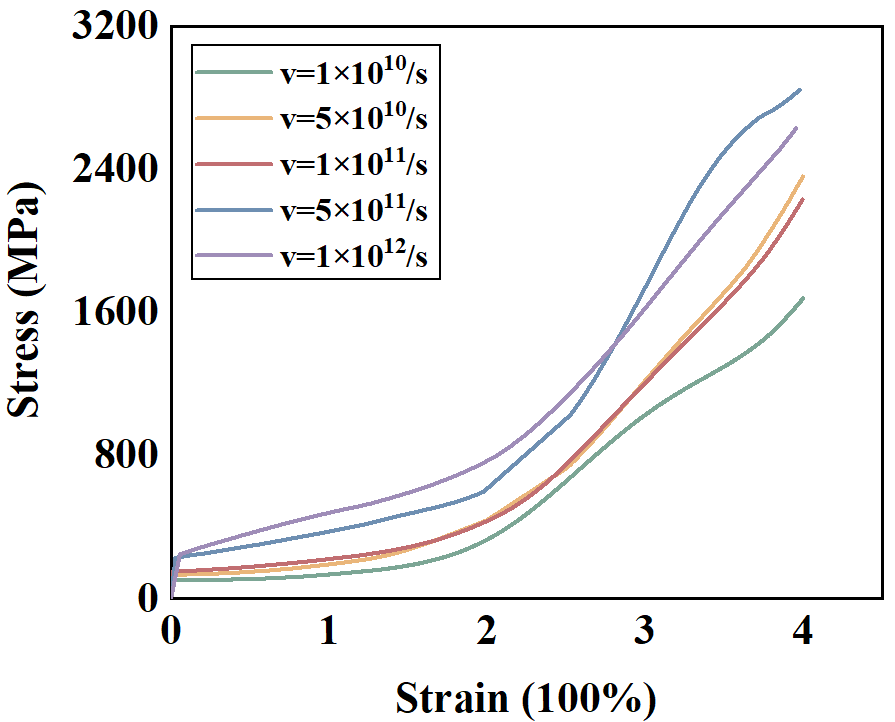
**

**Figure S15** Stress-Strain Curves corresponding to the composition 50-30-15-5.

**
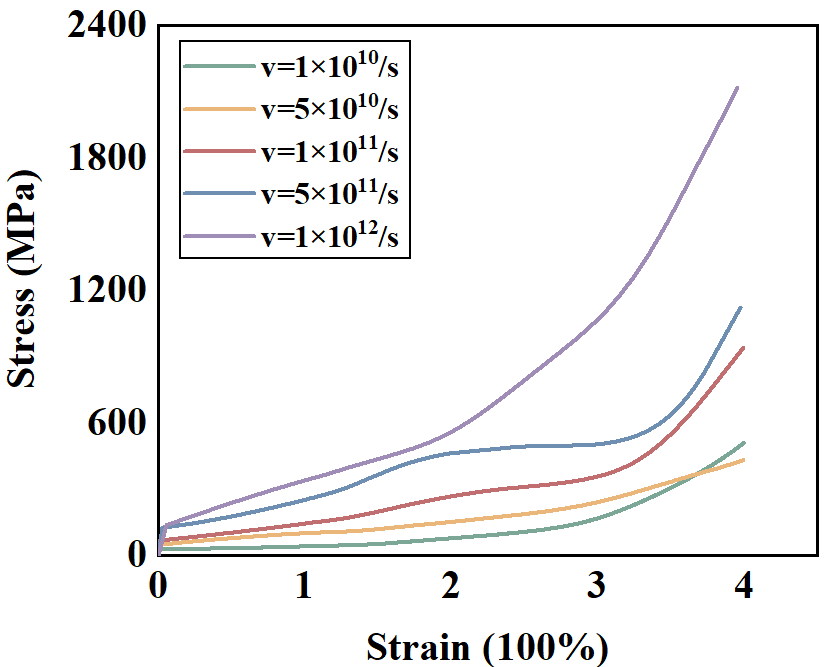
**

**Figure S16** Stress-Strain Curves corresponding to the composition 10-80-5-5.

**
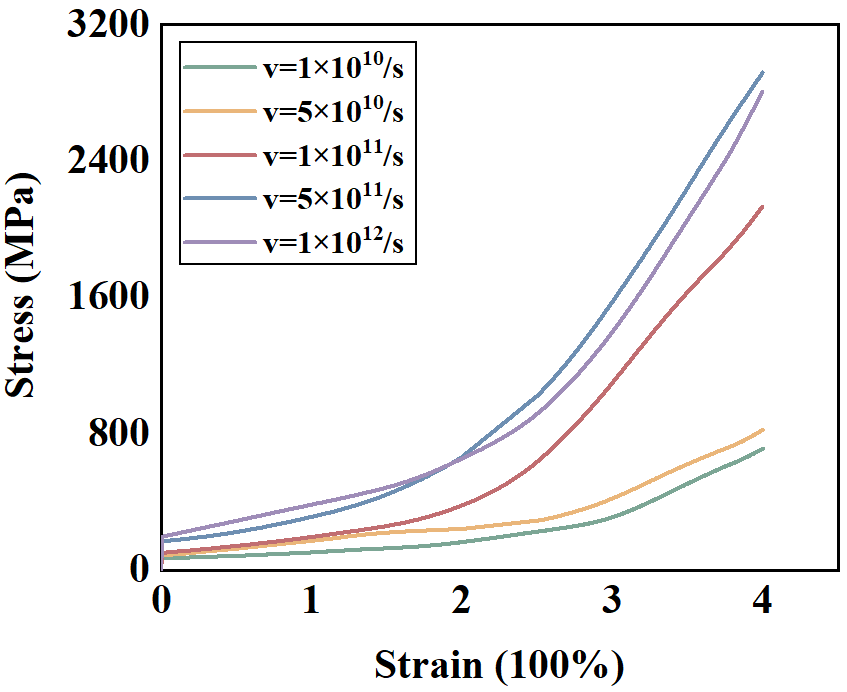
**

**Figure S17** Stress-Strain Curves corresponding to the composition 20-70-5-5.

**
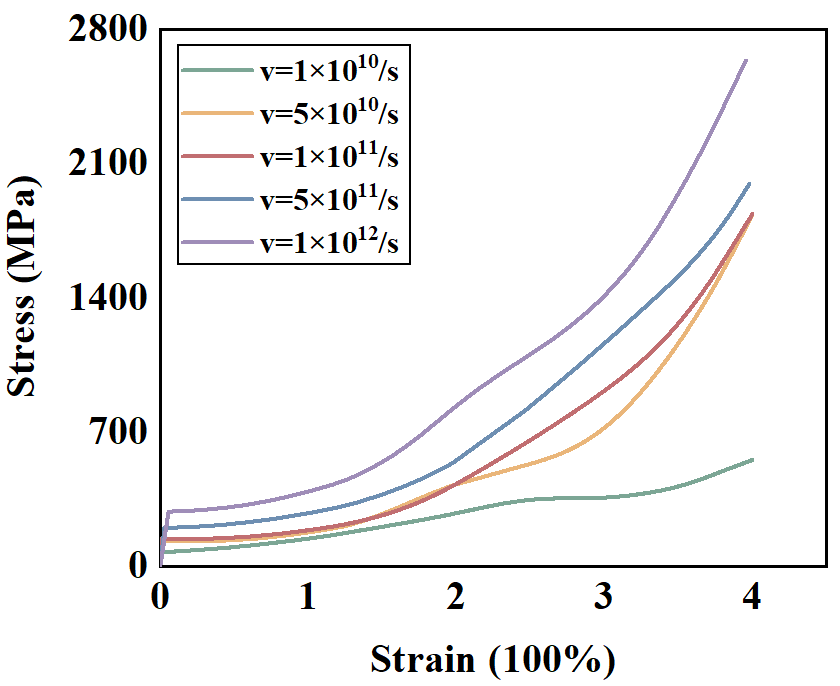
**

**Figure S18** Stress-Strain Curves corresponding to the composition 30-60-5-5.

**
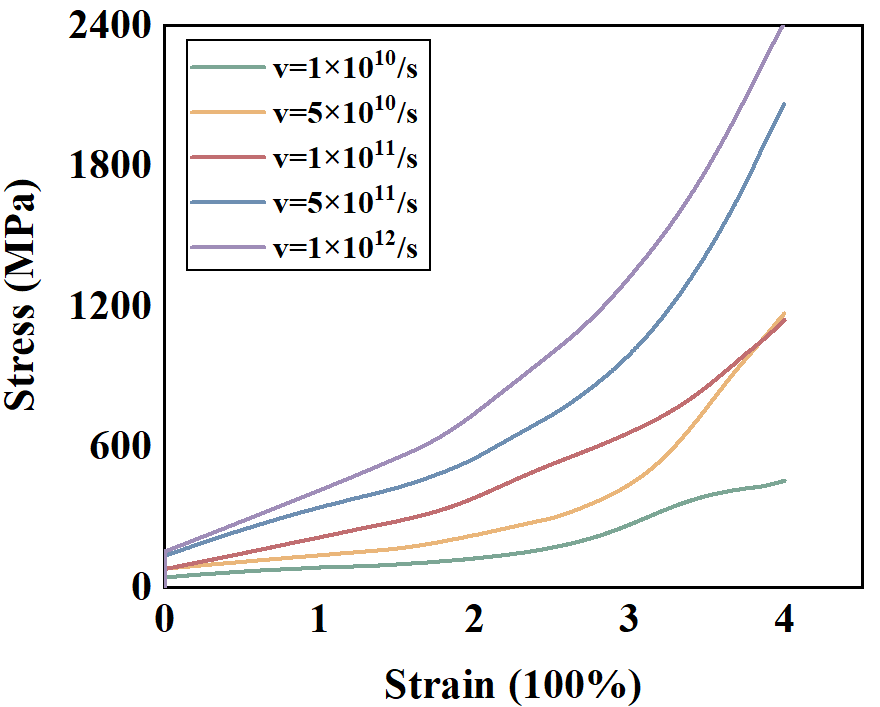
**

**Figure S19** Stress-Strain Curves corresponding to the composition 40-50-5-5.

**
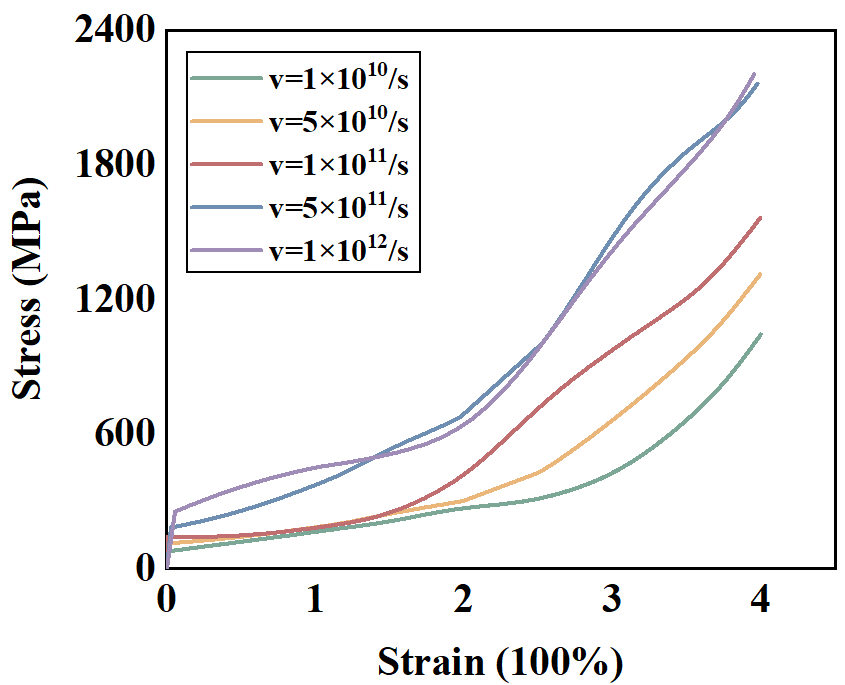
**

**Figure S20** Stress-Strain Curves corresponding to the composition 50-40-5-5.

**Section2. Experimental Section**

Three different commercial grades of SSBR were selected for compounding. During the mixing process, SSBR and other additives were sequentially added into an open two-roll mill according to the formulation ratios and mixed thoroughly under appropriate temperature and shear conditions until a homogeneous blend was achieved. The rubber was then placed into a rotorless rheometer to record the curing curve and determine the optimum curing time. Subsequently, the material was vulcanized using a flat-plate vulcanizing press according to the optimum curing time. After vulcanization, the samples were removed and cooled to room temperature for further testing.

According to the GB/T 528-2009 standard, the vulcanized sheets were cut into dumbbell-shaped specimens. The specimens were mounted on the grips of a universal tensile testing machine, and five tensile rates—100 mm/min, 200 mm/min, 300 mm/min, 400 mm/min, and 500 mm/min—were applied. During each test, both stress and strain data were continuously recorded. In total, fifteen stress-strain curves were obtained. The molar fractions of the four structural units (styrene, 1,2-butadiene, cis-1,4-butadiene, and trans-1,4-butadiene) for the three SSBR grades are listed in Table S2.

**Table S2** Structural unit compositions of SSBR used for model fine-tuning (mol%)

| No. | Styrene Content | 1,2-Butadiene Content | Cis-1,4-Butadiene Content | Trans-1,4-Butadiene Content |
| --- | --- | --- | --- | --- |
| 1 | 10 | 40 | 25 | 25 |
| 2 | 23 | 61 | 8 | 8 |
| 3 | 20 | 55 | 15 | 10 |

**Section3. Uncertainty Analysis of Inverse Design Predictions and Additional Experimental Validation**

To further assess the reliability of large-scale compositional predictions in the inverse design stage, an uncertainty analysis was performed for all inverse-designed candidate compositions based on the five-seed fine-tuning and uncertainty quantification framework described in the main text. Specifically, under identical pre-trained weights, data partitioning schemes, and network architectures, the complete two-stage fine-tuning procedure was repeatedly executed using different random seeds, resulting in multiple independently fine-tuned sub-models. For each inverse-designed candidate composition, stress predictions were obtained from all sub-models, and the standard deviation of these predictions was adopted as an uncertainty metric to quantify the degree of model disagreement at that compositional point. The degree of extrapolation of each inverse-designed candidate relative to the experimental data was quantitatively characterized using the Euclidean distance in the structural unit composition space to the nearest experimental data point. Larger distances indicate that the corresponding candidate compositions are more likely to reside in sparse or uncovered regions of the experimental dataset. Figure S21A presents a scatter plot of the prediction uncertainty as a function of this distance, together with a binned median trend to highlight the overall variation. A clear tendency of increasing uncertainty with increasing distance can be observed, indicating that as candidate compositions progressively deviate from the experimental data coverage, prediction discrepancies among different sub-models become more pronounced, reflecting a gradual increase in model uncertainty in potential extrapolation regions.

To further facilitate interpretation, Figure S21B categorizes all prediction points into three regions (Near, Mid, and Far) according to their distances from the experimental data. Compared with the Near region, the Mid region exhibits a noticeably higher median uncertainty, suggesting increased prediction dispersion as compositions move away from the training distribution. Notably, the uncertainty distribution in the Far region does not show a further monotonic increase, and its median value is not significantly higher than that of the Mid region. This observation indicates that, for certain more distant compositional regions, the model can still maintain a moderate level of predictive stability. Overall, these results suggest that the PENN framework does not merely rely on memorizing a limited number of experimental grades, but instead captures a continuous mapping between structural unit composition and mechanical response within a reasonable extrapolation range.

**
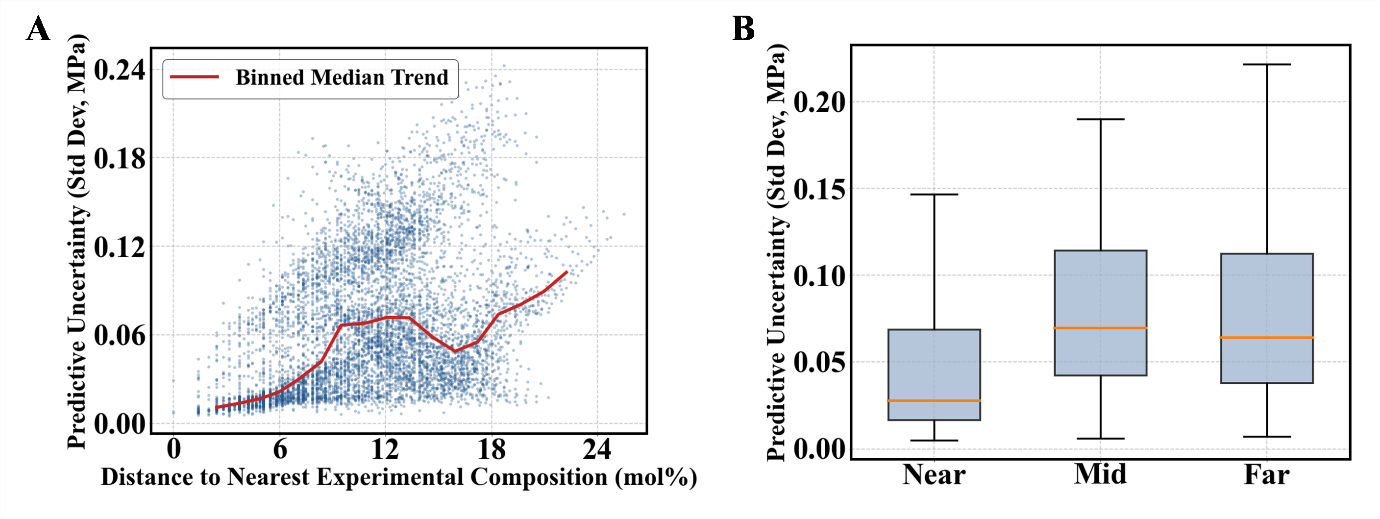
**

**Figure S21** A) Prediction uncertainty of inverse-designed candidate compositions as a function of distance to the nearest experimental composition. B) Distribution of prediction uncertainty grouped by distance-based regions (Near, Mid, and Far).

Building on the above uncertainty analysis, additional experimental validation was conducted to further evaluate the model’s predictive capability within a practically relevant extrapolation regime. Two commercial SSBR grades that were not involved in model training were selected for supplementary experimental testing. These materials exhibit noticeable differences in structural unit composition compared to the three SSBR grades used for training, while still falling within the compositional space explored by the inverse design procedure. The corresponding structural unit contents are summarized in Table S3. Using the fine-tuned PENN model, stress-strain responses under identical loading conditions were predicted for these two new grades and compared with experimental measurements. As shown in Figure S22, the predicted curves show good agreement with the experimental data in terms of overall trend and stress level, particularly in the small and intermediate strain regimes. Although certain deviations remain at high strain, the overall predictive accuracy remains within an acceptable range.

**
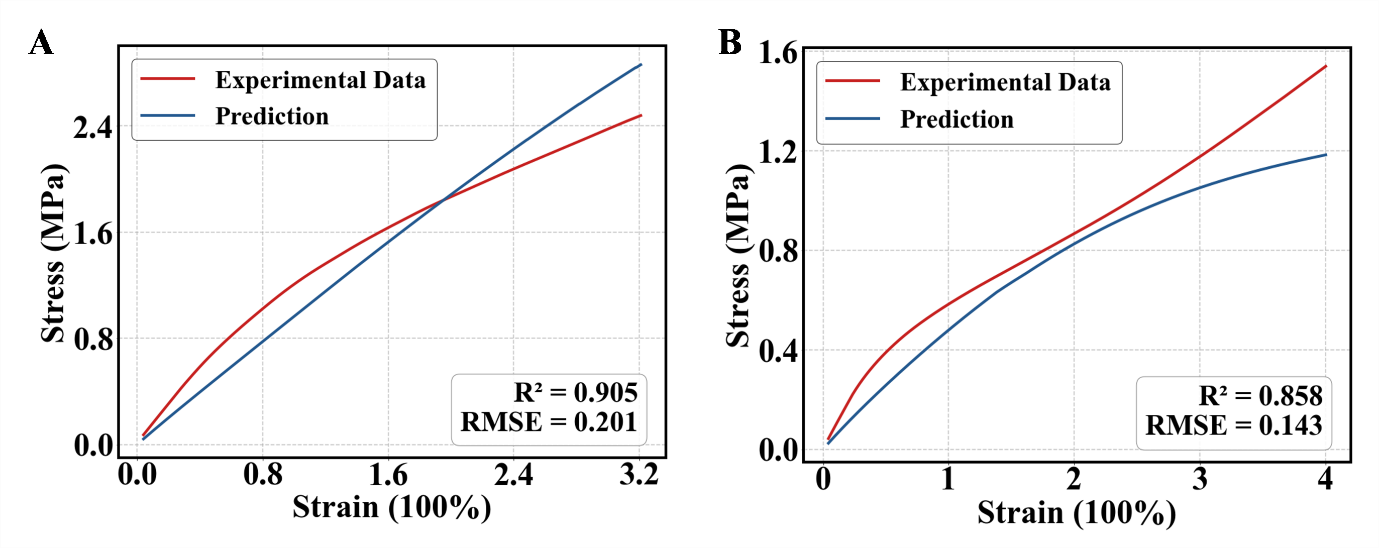
**

**Figure S22** Comparison between experimental stress-strain curves and PENN model predictions for two additional SSBR grades.

These supplementary experimental results demonstrate that, even under severe experimental data scarcity, the PENN model is capable of providing reasonably accurate predictions for new structural unit combinations within a practical extrapolation range. This finding further supports the credibility of the inverse design results and indicates that the model has learned a structure-property relationship with a certain degree of generalization, rather than simply memorizing a small number of known SSBR grades. It should be emphasized that the model is not intended to cover the entire unknown SSBR compositional space; however, within the structural unit variation ranges discussed in this work, it is able to deliver reliable predictive performance.

**Table S3** Structural unit compositions of additional SSBR grades for experimental validation (mol%)

| No. | Styrene Content | 1,2-Butadiene Content | Cis-1,4-Butadiene Content | Trans-1,4-Butadiene Content |
| --- | --- | --- | --- | --- |
| 1 | 35 | 40 | 7 | 18 |
| 2 | 20 | 68 | 10 | 2 |

**Section4. Algorithmic implementation (pseudocode) of the PENN framework**

**Pseudocode S1.** Yeoh-3 uniaxial stress formulation

function Yeoh_Uniaxial_Stress(C_params, λ):

# C_params = [C1, C2, C3], C1,C2,C3 ≥ 0

# λ: stretch ratio

I1 = λ² + 2 / λ

I1_bar = I1 − 3

# Derivative of strain energy with respect to I1

dW_dI1 = C1

+ 2 · C2 · I1_bar

+ 3 · C3 · (I1_bar)²

# Cauchy stress under uniaxial tension

σ = 2 · dW_dI1 · (λ² − 1 / λ)

return σ

**Pseudocode S2.** Bayesian optimization for selecting network hyperparameters

function MapToPowerOfTwo(x):

return 2 ^ floor(x)

function BuildPENN(dense_units1, dense_units2, learning_rate):

# two hidden layers with ReLU

# output: Yeoh parameters [C1, C2, C3] constrained non-negative (softplus)

# stress is computed via embedded Yeoh-3 uniaxial formulation

return compiled_model

function Objective(dense_units1, dense_units2, batch_size):

# 1) map continuous proposals to practical discrete settings

u1 = MapToPowerOfTwo(dense_units1)

u2 = MapToPowerOfTwo(dense_units2)

bs = MapToPowerOfTwo(batch_size)

# 2) build model with given hyperparameters

model = BuildPENN(u1, u2, learning_rate)

# 3) train with early stopping and learning-rate reduction on validation loss

Train(model, X_train, y_train,

epochs,

batch_size = bs,

validation_data = (X_test, y_test),

callbacks = {EarlyStopping, ReduceLROnPlateau})

# 4) evaluate and return R² on validation set (to be maximized)

y_pred = Predict(model, X_test)

return R2(y_test, y_pred)

# Search space (continuous variables; discretized inside Objective)

pbounds:

dense_units1 ∈ [3, 7] # mapped to 2^3 … 2^7

dense_units2 ∈ [3, 7] # mapped to 2^3 … 2^7

batch_size ∈ [5, 9] # mapped to 2^5 … 2^9

# Bayesian optimization loop (as implemented by the bayes_opt package)

Initialize BayesianOptimizer with Objective and pbounds

Run BayesianOptimizer to maximize Objective

Return the best observed (dense_units1, dense_units2, batch_size) and its validation R²

**Pseudocode S3.** Physics-Embedded Neural Network with Yeoh-3 embedding

function PENN_Yeoh3(X):

# X = [Styrene, 1,2-Bd, cis-1,4-Bd, trans-1,4-Bd, strain_rate, strain]

# Shared feature extraction

h = DenseLayer(X)

h = DenseLayer(h)

# Yeoh parameter heads (non-negative constraint)

C1 = Softplus(Dense(h))

C2 = Softplus(Dense(h))

C3 = Softplus(Dense(h))

C_params = [C1, C2, C3]

# Convert strain to stretch ratio

λ = 1 + strain

# Physics-based stress computation

σ_pred = Yeoh_Uniaxial_Stress(C_params, λ)

return σ_pred

**Pseudocode S4.** Two-stage transfer learning from MD to experimental data

# Pretraining on MD data

θ_MD = Train(PENN_Yeoh3, MD_dataset)

# Initialize experimental model

θ_exp ← θ_MD

# Stage A: parameter-head alignment

Freeze(shared_layers)

Unfreeze(Yeoh_parameter_heads)

Optimize(θ_exp, Experimental_dataset)

# Stage B: joint fine-tuning

Unfreeze(all_layers)

Optimize(θ_exp, Experimental_dataset)

return θ_exp

**Pseudocode S5.** Inverse design via systematic exploration of candidate compositions

function BuildCompositionSpace(st_range,bd12_range,cis14_range,

trans14_range,strain_rate_fixed,strain_fixed, tol):

# Input:

# st_range, bd12_range, cis14_range, trans14_range: predefined mol% ranges (step = 1 mol%)

# strain_rate_fixed, strain_fixed: fixed loading condition used for inverse design

# tol: tolerance for enforcing the sum-to-100 constraint

# Output:

# C: table of feasible candidate compositions with fixed loading condition

Initialize empty list C

for s in st_range:

for b12 in bd12_range:

for bc in cis14_range:

for bt in trans14_range:

total = s + b12 + bc + bt

if abs(total - 100) < tol:

Append [s, b12, bc, bt, strain_rate_fixed, strain_fixed] to C

return C

function PredictStressOnSpace(finetune_model, C):

# Input:

# finetune_model: fine-tuned PENN model used for inference

# C: candidate composition table produced by BuildCompositionSpace

# Output:

# C_pred: same table augmented with predicted stress

X = Extract columns [styrene, butadiene_12, butadiene_cis, butadiene_trans, strain_rate, strain] from C

y = finetune_model.predict(X)

Attach y to C as a new column "stress"

return C

**Section5. Influence of Hyperparameters on PENN Performance**

To further clarify the influence of individual hyperparameters on the performance of the PENN, an additional visualization of the Bayesian optimization results is provided (Figure S23). Unlike the joint representation shown in the main text, where multiple hyperparameters are simultaneously encoded in a single plot, this figure disentangles their effects by examining each hyperparameter independently.

Figure S23 presents the relationship between the R^2^ and three key hyperparameters, namely the number of units in the first dense layer (Dense Units_1), the number of units in the second dense layer (Dense Units_2), and the batch size. For each hyperparameter, all evaluated configurations obtained during Bayesian optimization are shown as semi-transparent scatter points, reflecting the overall performance distribution and variability. Superimposed on these data, a solid line indicates the best achievable R^2^ value at each discrete hyperparameter setting, while the red star highlights the global optimum identified across the corresponding search space.

**
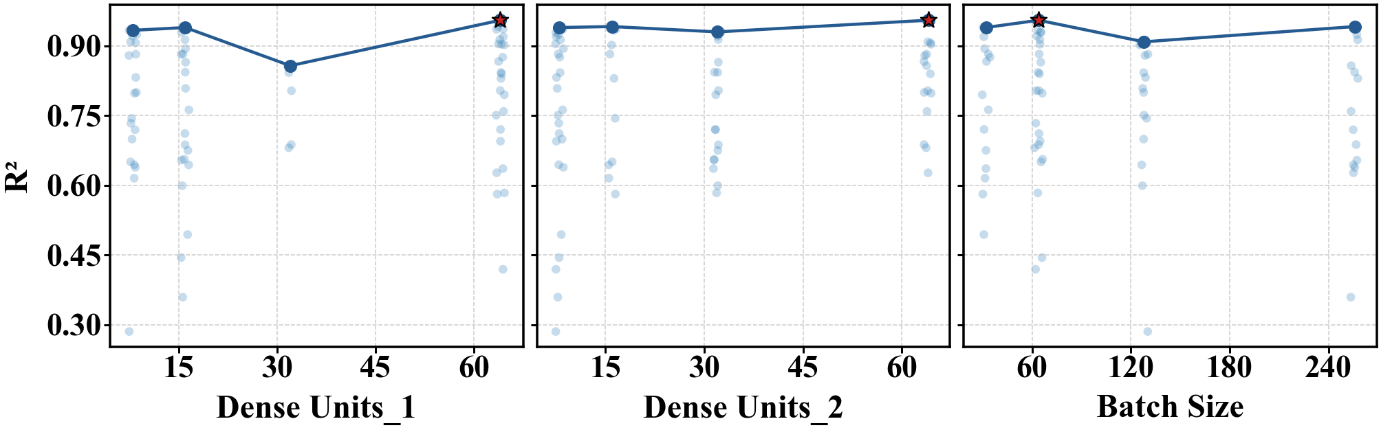
**

**Figure S23** Influence of individual hyperparameters on the performance of the PENN obtained from Bayesian optimization.

This visualization strategy provides a clearer and more intuitive view of the hyperparameter selection process. By presenting each hyperparameter separately, Figure S23 allows the influence of network width and batch size on model performance to be more clearly observed, avoiding the overlap and visual complexity inherent in the combined representation. The best-performing configuration at each discrete hyperparameter value is explicitly highlighted, enabling straightforward identification of favorable parameter ranges. Overall, Figure S23 complements the main-text analysis by offering a more transparent illustration of how individual hyperparameters affect the performance of the PENN.
